# Editorial: Innovative studies in organized helping: transforming relations, emotions and referents through sequentially structured practices

**DOI:** 10.3389/fpsyg.2024.1408009

**Published:** 2024-04-12

**Authors:** Claudio Scarvaglieri, Peter Muntigl, Eva-Maria Graf

**Affiliations:** ^1^Department of German Language and Literature, University of Lausanne, Lausanne, Switzerland; ^2^Department of Translation, Interpreting and Communication, Ghent University, Ghent, Belgium; ^3^Department of English and American Studies, Klagenfurt University, Klagenfurt, Austria

**Keywords:** transformative sequences, organized helping, emotions, relationships, referents, communicative practices

Communication is central to solving (inter-)personal problems in the helping professions (psychotherapy, counseling, coaching, helplines, mediation etc.). Peräkylä's (2019) paper on “transformative sequences” in psychotherapy has argued that important change events occur in three dimensions that are grounded in conversational practice: relations, referents and emotions. For example, a “good” relationship between the help provider and the client bears a significant, positive relation to the outcome of the process (Norcross and Lambert, 2018). However, the majority of the existing research mainly relies on quantitative methods, rather than a detailed examination of the specifics of communicative events and how this can develop an in-depth understanding of how transformative sequences are achieved through the talk and conduct of help-providers and clients. The 13 contributions to this Research Topic rely on qualitative, interaction-focused methods to describe and understand transformative sequences as an organized societal practice. These works examine transformative sequences in relation to four partly overlapping themes, namely emotion, relationships, referents and communicative practices.

## Emotions

The display, processing and transformation of emotions is a core aspect of many helping professions (Muntigl, 2023). Working toward “emotionography” – “a comprehensive study of emotions as they occur naturally” – Hepburn and Potter documented child protection helpline cases of callers displaying emotion, such as crying or laughing. They discuss how participants orient to emotions as stance displays and how emotions contribute to interactive practices, such as laughing to manage resistance to advice. Emotions are thus presented as emerging in interaction and contributing to a transformation of the initial interactive situation that makes this situation manageable for both participants. Telephone data were also investigated by Slembrouck et al. The authors analyzed COVID-19 contact-tracing calls and focused on the fact that contact tracers need to transform their clients' emotions during the call. Their data illustrate that telephone agents use humor and other mitigating strategies to be able to communicate with their clients in a productive way, which creates the basis for providing advice about future behavior. Yu et al. investigated the usage of words and emojis in Hong Kong discussion forums during the pandemic to express concern, ask for information, and engage with others. They argue that, while they do serve to communicate emotions, emojis also carry pragmatic meanings and illocutionary force and can alter the illocutionary force of the preceding text.

## Relationships

Many contributions to this Research Topic also demonstrated how the relationship between the participants is managed and transformed, as the relationship is one of the principal factors contributing to change in the client. Investigating psychoanalytic individual therapy, Herrera et al. relied on the concept of “moments of meeting” (Stern, 2009) and discussed how such an interactive moment that transforms the relationship between therapist and client is sequentially accomplished through a practice that the authors term “co-animation.” In this interactive sequence, the client expressed and exercised her own agency and assumed an active role that is ratified and supported in the therapist's response. Such moments of meeting thus transformed the participants' relationship as well as the patient's perspective on herself and her agency. Muntigl and Scarvaglieri provided an overview of the linguistic research on the relationship in psychotherapy, discussing affiliation and alignment between therapist and patient as well as empathy and sequences intended to repair a strained relationship. Adding to this, Muntigl and Horvath analyzed *observer-perspective* questions in couples therapy that elicit the clients' perspectives on the thoughts and intents of the partners present. The authors identified four kinds of changes that questions in this setting can promote, which include the introduction of new relational options and progress toward relational optimism. Relationship management is also at the center of Kabatnik's contribution, which focused on communication in messenger-supported group therapy. The author's study demonstrates how participants build and manage relationships both in-group and with people outside of the group. In Kabatnik's data, the therapist acts as an important agent of change, first making clients aware of their current state and establishing a comparison with a target state, which then makes it possible to record change. Many studies have discussed communicative practices that not only manage relationships but also solve them. Jautz et al., for example, investigated agenda-setting in coaching as one of the core activities designed to structure the entire ensuing coaching process. They show that this activity is often conducted at the start of a coaching dyad and is crucial for establishing a working relationship between the participants and that both coach and client frequently orient themselves toward the importance of the relationship while setting the agenda.

## Referents

Wahlström investigated the varied usage of pronoun references in the first sessions of psychotherapy at a university training clinic. This author's work shows that therapists in initiative turns usually use the second person singular when addressing the patient, while patients often react using “zero-person” constructions that do not identify a subject of an action and instead portray experiences as common to people in general. Reacting to this, therapists regularly use a combination of zero and active person references to show empathy toward the client while at the same time inviting them to take an agentic position toward their own experience.

## Communicative practices

Analyzing transformative communicative practices is another major theme of the contributions to this Research Topic. Dionne et al. investigated conversational data from business coaching and focused on practices in which clients resist the interactional constraints placed by wh-questions. By examining various resistance practices, the researchers demonstrate that clients use these to transform the course of action projected by wh-questions and thereby steer the interactional process in a different direction more suitable to their current needs. In a related study, Moos and Spranz-Fogasy examined questions asked by coaches immediately after a rephrasing or relocating action. As the data show, such questions not only prompt the client to respond in an explicit or implicit way, but also support self-reflection, which the authors consider one of the factors supporting change and transformation in the client. Based on data from psychoanalytic psychotherapy, Franzen et al. focused on the recording situation and how patients orient to being recorded. They argue for the need for a deeper theoretical understanding of the observer paradox in therapy (cf. Labov, 1972) and show that therapists can use patients' orientation to the recording situation to initiate and support patients' self-exploration and to support change (cf. Pawelczyk and Graf, 2019; Scarvaglieri, 2020). Tauroginski et al. worked with data from psychoanalytic couples therapy and focused on complaints, which in this setting is a frequently recurring activity. The authors describe in detail how complaints are composed and delivered and they argue that the specific way of formulating complaints can be used to glean information about the nature of the spouses' quarrels and about their personality structure.

The articles in this Research Topic thus demonstrate how emotions, relations and referents are transformed in the different helping professions investigated and what communicative practices are frequently employed to achieve this aim. This Research Topic can provide important insights into transformative communication that are of relevance to researchers from a variety of academic backgrounds (including linguistics, psychology, and sociology) and to practitioners from the institutional settings examined.

## Author contributions

CS: Writing – original draft, Writing – review & editing. PM: Writing – review & editing. E-MG: Writing – review & editing.
